# Zoonoethics and Inclusive One Health Governance for H5N1 Panzootic: From Animal Culling to Co-responsibility

**DOI:** 10.1093/phe/phag002

**Published:** 2026-03-05

**Authors:** Juan Alberto Lecaros, Jeyver Rodriguez

**Affiliations:** Observatory of Bioethics and Law, Santiago de Chile, Chile; Institute of Sciences and Innovation in Medicine, Faculty of Medicine, Clínica Alemana Universidad del Desarrollo, Santiago de Chile, Chile; Center for Socio-Legal, Criminological, and Ethical Research (CISCE), Universidad Central de Chile, Santiago de Chile, Chile; Cape Horn International Center, Puerto Williams, Chile

## Abstract

The global spread of highly pathogenic avian influenza (H5N1) clade 2.3.4.4b has evolved into a multispecies panzootic that disrupts conventional boundaries between human, animal and environmental health systems. Using Brazil’s response as an illustrative case, this article argues that prevailing containment strategies—particularly mass culling—remain ethically insufficient and practically misaligned with the ecological complexity of H5N1 transmission. To clarify the normative foundations of an alternative approach, we introduce a theoretical framework grounded in zoonoethics and global ecological bioethics, emphasizing multispecies justice, relational vulnerability, intercultural and community engagement and co-responsibility. We then apply this framework to evaluate the limitations of reactive biosecurity paradigms and to outline multispecies-sensitive One Health governance guidelines. The analysis demonstrates that effective and legitimate panzootic response requires moving beyond biomedical and anthropocentric models toward anticipatory, inclusive and ethically grounded governance capable of addressing structural drivers such as biodiversity loss, land-use change and the erosion of Indigenous territorial protections. We conclude by discussing how the H5N1 panzootic represents a ‘perfect storm’ that demands not only improved preparedness, but a reconceptualization of One Health as an ethical and political project of multispecies cohabitation in an era of planetary instability.

## Introduction

Over the past three decades, highly pathogenic avian influenza (HPAI) has shifted from a relatively contained animal health concern to a persistent, global multispecies risk. Since the mid-1990s, the repeated mutation of low-pathogenic H5 and H7 viruses within intensive poultry production systems has generated viral lineages with unprecedented ecological reach and evolutionary plasticity, enabling sustained circulation across species and ecosystems (Chrzastek *et al.*, 2025; Krammer *et al.*, 2025; Lambertucci *et al.*, 2025; Peacock *et al.*, 2025). This transformation represents not only an epidemiological turning point but also an ethical one, foregrounding questions of human responsibility for the socio-ecological conditions that enable large-scale and recurrent multispecies harm.

Among these lineages, H5N1 clade 2.3.4.4b has emerged as a paradigmatic case of a contemporary panzootic. Since early 2024, this clade has expanded rapidly across the Americas, affecting a wide range of hosts including poultry, migratory and resident wild birds, marine mammals, terrestrial mammals, and, in isolated cases, humans (Garg *et al.*, 2025; Kamel *et al.*, 2025; Bellido-Martín *et al.*, 2026). These developments challenge conventional assumptions that zoonotic risk can be effectively contained within discrete species, sectors or territorial boundaries. Instead, they reveal a landscape of health governance in which biological, ecological and political domains are deeply entangled.

The geographic scale of this expansion is continental. According to the Pan American Health Organization (PAHO, 2025, p. 1), H5N1 spread ‘through the main migratory routes of waterfowl to North America and, in 2022, to Central and South America,’ illustrating how hemispheric flyways function as ecological infrastructures that connect distant ecosystems and redistribute risk across regions. These migratory networks, stretching from the Arctic to Patagonia, operate not merely as biological pathways but as ecological complexity corridors shaped by climate variability, land-use change, global food systems and governance regimes. Comparable dynamics have been observed in Europe, where recurrent outbreaks of H5N1 clade 2.3.4.4b—most notably in Germany—have persisted despite advanced veterinary infrastructures and coordinated regional responses, underscoring the limits of technologically driven containment strategies (Friedrich-Loeffler-Institut, 2025; Krammer *et al.*, 2025).

At the international level, dominant surveillance and reporting frameworks further complicate ethical assessment. The World Organisation for Animal Health (WOAH, 2025, p. 8) defines the impact of HPAI outbreaks primarily through aggregated indicators such as ‘number of outbreaks, associated cases and losses,’ where losses include animals that have died, been killed, or disposed of during response measures. This aggregation collapses distinct forms of harm—disease-related mortality, preventive killing, and disposal—into a single metric, thereby obscuring both the scale and the moral significance of multispecies suffering. Framing animal deaths predominantly as ‘poultry losses’ reinforces an anthropocentric logic that prioritizes trade continuity and biosecurity efficiency while marginalizing ethical considerations related to animal vulnerability, ecological integrity and distributive justice.

These limitations point to a deeper tension within prevailing One Health governance models. Although One Health principles emphasize the interdependence of human, animal and environmental health, its operationalization often remains anchored in reactive biosecurity, sectoral silos and biomedical risk management. Animals are frequently treated as reservoirs, vectors, or economic units, rather than as beings embedded in shared complex socio-ecological systems.

A growing body of research indicates that zoonotic emergence cannot be separated from large-scale anthropogenic transformations reshaping ecosystems at planetary scale (Allen *et al.*, 2017; Carlson *et al.*, 2025; Morris and Wada, 2025; Silva and Soares, 2025). Climate change, deforestation, wetland degradation, industrial livestock production, environmental crime and the global wildlife trade destabilize habitats and disrupt historical migratory routes, generating novel interfaces between wildlife, domestic animals and human populations. These dynamics are particularly pronounced in regions such as the Brazilian Amazon, where accelerated land-use change, habitat fragmentation and extractive economies erode ecological resilience and intensify multispecies contact zones.

Within this context, migratory routes can be understood not only as mechanisms of pathogen dispersal but as indicators of systemic vulnerability within interconnected socio-ecological systems (Jindal *et al.*, 2025; Lambertucci *et al.*, 2025; Plaza *et al.*, 2025). The same processes that sustain biodiversity, nutrient cycling, and long-distance connectivity now also facilitate the continental circulation of pathogens. Rising temperatures, altered phenological cycles, and increasing light and noise pollution further reshape migratory behaviors, concentrating animals at fewer stopover sites and amplifying opportunities for cross-species viral transmission. Recent events in the Global South make this entanglement particularly visible. In Chile, for example, HPAI emerged in late 2022, causing mass mortality among wild birds, poultry, marine mammals and a confirmed human case, followed by detections in Antarctica (Pardo-Roa *et al.*, 2025). Such developments unsettle conventional boundaries between human, animal and environmental health, revealing zoonotic risk as a manifestation of broader ecological and governance failures.

Against this backdrop, this article examines Brazil’s response to the H5N1 panzootic as an illustrative case. Brazil occupies a strategic position within global agri-food systems as one of the world’s largest poultry exporters and a key factor in regional One Health initiatives. Yet its response to H5N1 has relied predominantly on mass culling and a techno-fix model of reactive biosecurity. While these measures aim to secure trade and contain immediate risk, they raise significant ethical concerns regarding multispecies justice, epistemic inclusion and responsibility for structural drivers of zoonotic emergence.

The central aim of this article is to critically assess the normative and epistemic limitations of such reactive strategies and to argue for a more anticipatory, relational and ethically grounded form of One Health governance. Drawing on zoonoethics and global ecological bioethics, we propose a framework that foregrounds interspecies justice, relational vulnerability, co-responsibility and epistemic pluralism. By situating Brazil’s response within a transcontinental and multispecies perspective, the article contends that panzootic events such as H5N1 demand governance approaches that move beyond surveillance and culling toward ecologically informed, ethically robust and publicly accountable forms of health governance.

The article proceeds as follows. ‘Case Study: Brazil’s Response to H5N1 and the Limits of Reactive Biosecurity Model’ section presents Brazil’s response to H5N1 within the broader panzootic dynamics unfolding across the Americas. ‘Theoretical Background: Zoonoethics and Global Ecological Bioethics’ section introduces zoonoethics and global ecological bioethics as complementary frameworks for rethinking One Health beyond reductionist approach to biosecurity. ‘Beyond Culling and Reactive Biosecurity Responses’ section critically examines mass culling and related containment strategies. ‘Ethical Guidelines for Inclusive and Context-sensitive One Health Governance’ section outlines ethical guidelines for inclusive and multispecies-sensitive One Health governance. ‘Discussion: A Perfect Panzootic Storm for One Health Governance’ section discusses the H5N1 panzootic as a ‘perfect storm’ for public health ethics, and ‘Conclusion’ section concludes by summarizing the implications of this analysis for ethically grounded pandemic preparedness.

## Case Study: Brazil’s Response to H5N1 and the Limits of Reactive Biosecurity Model

Brazil has positioned itself as a regional advocate of integrated health governance through its endorsement of the São Paulo Declaration on One Health, which emphasizes equity, environmental stewardship, multisectoral coordination, and the integration of human, animal and ecosystem health (Soeiro-Barbosa *et al.*, 2025). In principle, the Declaration articulates a vision of One Health that aligns closely with emerging ethical critiques of anthropocentric and technocratic public health models. Yet the trajectory of Brazil’s response to the H5N1 panzootic reveals a significant gap between these normative commitments and the operational logic guiding outbreak management. This gap provides a critical lens through which to assess the limitations of prevailing biosecurity governance.

As H5N1 circulated along migratory flyways connecting coastal, Amazonian and wetland ecosystems, Brazil’s response remained largely framed as a technical problem of containment rather than as a manifestation of deeper socio-ecological vulnerability. Federal authorities moved quickly to activate emergency protocols and inter-agency coordination, but the dominant framing emphasized trade security, sanitary certification and the continuity of agribusiness operations. In its official communications and self-reporting to international bodies, Brazil underscored the safety of poultry products and the effectiveness of stamping-out measures, presenting the outbreak primarily as a manageable biosecurity incident rather than as a multispecies public health challenge (WOAH, 2025). This orientation reflects what may be described as a techno-fix model of One Health governance: an approach that prioritizes technical control and sectoral coordination while leaving the structural drivers of zoonotic risk largely unaddressed.

Central to this model was the reliance on mass culling as the default instrument of disease control. While culling is widely accepted within international veterinary frameworks as an emergency response to HPAI, its ethical and ecological implications are rarely subjected to sustained public deliberation (Degeling *et al.*, 2016; Lederman *et al.*, 2021; Anderson and Reaser, 2024; Lerner, 2025). In Brazil, as in many other contexts, culling was treated as an unquestioned necessity, implemented rapidly to restore disease-free status and reassure international markets. However, when deployed in isolation from expanded wildlife surveillance, ecological monitoring and community engagement, such measures fail to address the broader processes that enable viral persistence and evolution. Moreover, the focus on commercial poultry obscures the multispecies dimensions of the panzootic, including mortality among wild birds and mammals, which often remains underreported or falls outside formal surveillance frameworks.

These limitations are not unique to Brazil. Similar patterns have been observed across regions affected by H5N1, where early and decisive elimination of animals within production chains is coupled with risk communication strategies aimed primarily at economic reassurance (Bruno *et al.*, 2025; Mena *et al.*, 2025). What the Brazilian case makes particularly visible, however, is how such responses intersect with long-standing structural vulnerabilities. Extensive land-use change, deforestation, mining, monoculture expansion and intensive livestock production—especially in the Amazon region—have eroded ecological buffers and intensified contact zones between wildlife, domestic animals and human populations (Rohr *et al.*, 2019; Mahon *et al.*, 2024; Carlson *et al.*, 2025).

These transformations displace Indigenous and rural communities, compress wildlife into fragmented habitats, and generate precisely the interfaces at which zoonotic spillover becomes more likely. Viewed through this lens, H5N1 cannot be understood solely as a virological event; it emerges as a symptom of what has been described as structural pathogenesis (Sell and Williams, 2020), in which political–economic choices and environmental degradation co-produce epidemic risk.

Institutional fragmentation further complicates Brazil’s capacity to govern such risks ethically and effectively. Responsibility for surveillance and response is distributed across multiple agencies with distinct mandates: the Ministério da Agricultura e Pecuária (MAPA) oversees poultry and livestock health; the Ministério da Saúde (MS) addresses human health surveillance; ICMBio monitors wildlife within protected areas; IBAMA enforces broader environmental and wildlife regulations; and state-level agencies conduct field operations. While this functional specialization reflects the complexity of One Health governance, coordination across these bodies is uneven, and reporting pathways are often opaque to local stakeholders (Aguilera *et al.*, 2025). As a result, communities, fishers, farmers and even municipal authorities frequently lack clarity about where and how to report dead birds or suspected outbreaks. This uncertainty delays information flow, undermines early detection and creates gaps in multispecies surveillance—precisely the vulnerabilities that One Health is intended to mitigate.

Beyond questions of efficiency, this fragmentation raises ethical concerns about accountability, inclusion and multispecies justices. Indigenous knowledge systems and the experiential expertise of smallholder farmers—who are often closest to local ecological change—remain marginal to formal surveillance and decision-making processes. This exclusion reflects deeper epistemic hierarchies within national governance structures, in which scientific and technical expertise is privileged over local and Indigenous forms of knowledge. Such hierarchies not only weaken early warning capacities but also reproduce patterns of injustice in which those most exposed to ecological disruption have the least voice in shaping responses (Boudreau LeBlanc *et al.*, 2022, 2025). Similar critiques have been directed at international organizations, including WOAH, whose classificatory practices tend to frame animals primarily as epidemiological units or economic losses, reinforcing an instrumental view of non-human life (Johnson *et al.*, 2024).

The ethical implications of this governance model became particularly salient with Brazil’s first confirmed outbreak of H5N1 in commercial poultry in May 2025. This event marked a critical shift in the regional epidemiology of the virus and was described as ‘a sentinel event for cross-border preparedness,’ highlighting both its epidemiological significance and the limited capacity of existing frameworks to anticipate and manage multispecies risk across interconnected socio-ecological systems (Martins-Filho and Quintans-Júnior, 2025). While emergency measures were effective in containing the immediate outbreak, they did little to address the structural conditions that make such events increasingly likely and recurrent.

From an ethical perspective, the Brazilian case highlights the fragility of reactive biosecurity when it operates as a dominant mode of One Health governance, underscoring the need for governance frameworks that explicitly integrate ethical reflection into decision-making rather than treating it as ancillary to technical control (Degeling *et al.*, 2015; Johnson and Degeling, 2019). Emergency protocols prioritize speed and containment, often at the expense of transparency, ethical justification and long-term prevention (van Herten *et al.*, 2019; van Herten and Bovenkerk, 2021). Decisions involving large-scale animal killing are treated as technical imperatives rather than as morally contestable choices that distribute harms unevenly across species, communities and ecosystems. This approach externalizes ecological costs, displaces responsibility onto future outbreaks, and limits opportunities for adaptive learning. As the panzootic evolves and new host species become involved, the inadequacy of strategies that treat animals as expendable units within biosecurity systems becomes increasingly apparent.

In this sense, Brazil’s response is best understood not as an outlier but as emblematic case of broader tensions within global One Health governance. The case reveals how commitments to integration and sustainability coexist uneasily with institutional practices shaped by trade imperatives, centralized decision-making, and short-term crisis management. It also underscores the ethical stakes of continuing to rely on reactive containment in the face of structurally driven, multispecies risks (Celermajer and McKibbin, 2023). By examining Brazil’s response through the lenses of zoonoethics and global ecological bioethics, the following sections seek to clarify why prevailing biosecurity paradigms remain normatively insufficient and how One Health governance might be reoriented toward more anticipatory, inclusive and ecologically responsible forms of public health practice.

## Theoretical Background: Zoonoethics and Global Ecological Bioethics

This article adopts a primarily conceptual and normative orientation to clarify the ethical frameworks required for governing multispecies panzootics such as H5N1. Rather than proposing an abstract moral theory detached from practice, this section develops the ethical scaffolding necessary to evaluate existing One Health responses and to articulate alternative governance orientations. Zoonoethics and global ecological bioethics are presented here as complementary frameworks: the former foregrounds multispecies justice and relational vulnerability at sites of zoonotic emergence, while the latter provides a planetary, intergenerational horizon for ethical responsibility within dynamic and interconnected socio-ecological systems. Together, they offer the normative depth needed to address the ethical blind spots exposed by reactive biosecurity governance.

### Zoonoethics: Philosophical Foundations for Ethical One Health Governance

Zoonoethics emerges as a critical response to the structural and multispecies conditions that underlie zoonotic spillovers. As articulated by Rodriguez (2024, 2025), zoonoethics constitutes an ethical framework that calls for a reconfiguration of human responsibilities at the intersection of human, animal, plant and environmental health. Crucially, it extends ethical consideration beyond human populations to encompass companion, farmed animals, and wildlife, while also recognizing pathogens and viruses as elements of complex socio-ecological systems. Rather than treating animals as passive recipients of human intervention or as mere vectors of disease, zoonoethics conceptualizes them as beings embedded within networks of interdependence that structure vulnerability, exposure, and harm.

A central contribution of zoonoethics lies in its anticipatory, intercultural and multi-actor orientation (Rodriguez, 2025). In contrast to ethical approaches that respond to harm only after it has occurred, zoonoethics foregrounds responsibility, precaution and relational accountability before damage becomes irreversible. This anticipatory stance is particularly salient in the context of H5N1, where repeated outbreaks signal not isolated failures but patterned, multifactorial socio-ecological vulnerabilities. Zoonoethics thus provides conceptual tools for ethical reasoning under conditions of uncertainty, complexity, and cascading risk, where decisions taken in the present shape multispecies futures.

Zoonoethics is grounded in two intertwined normative concerns. First, it responds to the need for an ethical framework capable of enriching One Health governance beyond its predominantly biomedical and technocratic orientation. While One Health has successfully highlighted the interdependence of human, animal and environmental health, it has struggled to articulate robust ethical principles capable of guiding decision-making in contexts of conflict, uncertainty and power asymmetry. Zoonoethics addresses this gap by foregrounding interspecies justice, relational vulnerability and shared responsibility as core ethical commitments (Lecaros, 2016b).

Second, zoonoethics is rooted in an ecological awareness of the contemporary planetary crisis. Accelerating biodiversity loss, environmental crimes, climate change and socio-political instability are not external background conditions but constitutive drivers of zoonotic risk. From this perspective, zoonotic outbreaks such as H5N1 are not abnormalities but predictable outcomes of socio-ecological disruption. Zoonoethics, therefore, situates zoonotic governance within broader questions of ecological responsibility, biocultural sustainability and justice across species and generations (Bartlett and Uhart, 2024)

By insisting that zoonotic risk is structurally produced rather than biologically accidental (Ortiz-Millán, 2025), zoonoethics reframes public health ethics as a matter of collective stewardship rather than technical control. It challenges the tendency of outbreak governance to reduce ethical deliberation to questions of efficiency, cost–benefit analysis, or emergency necessity, and instead demands reflection on how human institutions, production systems and governance choices distribute vulnerability across multispecies communities.

### What Zoonoethics Is Not: Distinguishing It From Biosecurity Ethics and Biomedical One Health

Clarifying the normative scope of zoonoethics requires distinguishing it from related but more limited ethical approaches. First, zoonoethics is not an anthropocentric extension of traditional bioethics. While critiques such as those offered by Beever and Morar (2025) rightly caution against technocratic optimism within One Health, they mischaracterize zoonoethics as merely reproducing human-centered moral reasoning. By contrast, zoonoethics explicitly challenges anthropocentrism by recognizing animals as morally considerable beings embedded within shared socio-ecological systems. On this view, ethical deliberation cannot begin from the assumption that human interests automatically outweigh nonhuman harms. Second, zoonoethics cannot be reduced to an ethical framework narrowly confined to zoonotic diseases. Although it is attentive to context-specific dilemmas—such as culling practices, surveillance failures, or the exclusion of Indigenous knowledge in outbreak response—it is also inherently global in scope. Contemporary zoonotic risks are transboundary phenomena shaped by global production systems, trade regimes, land-use transformations, and climatic processes. Zoonoethics, therefore, operates across scales, linking situated ethical reasoning to questions of global ecological responsibility and climatic justice. Third, zoonoethics resists assimilation into forms of biosecurity ethics grounded primarily in narrow economic calculation and emergency management. Scientific expertise is indispensable for understanding pathogens and transmission dynamics, but zoonoethics insists that science alone cannot determine how harms, benefits and responsibilities ought to be distributed across species, communities and institutions. Practices such as mass culling, waste disposal, and movement restrictions are often normalized as technical necessities, evaluated mainly in terms of effectiveness. Zoonoethics introduces a critical normative layer that interrogates the values, power relations and assumptions embedded in such practices, particularly when they result in large-scale multispecies harm.

In this sense, zoonoethics not only complements ‘One Health,’ but also offers principles for a more systematic and comprehensive risk assessment and response. By foregrounding relational vulnerability, multispecies justice and epistemic pluralism, zoonoethics provides a framework for ethical governance capable of addressing the blind spots identified in critiques of One Health: its tendency to universalize Western scientific norms (Wabnitz *et al.*, 2025), instrumentalize non-human life (Coghlan and Coghlan, 2018; Coghlan *et al.*, 2021; Johnson *et al.*, 2024), and overlook the structural inequities that shape health outcomes (Egede *et al.*, 2024; Luna and Holzer, 2025).

### Global Ecological Bioethics: Planetary Horizons and Reflexive Governance

While zoonoethics articulates ethical responsibilities at multispecies interfaces, global ecological bioethics offers a broader ethical grounding horizon for governance by situating health within planetary socio-ecological systems. From its inception in the work of Van Rensselaer Potter (Potter, 1988), global bioethics has emphasized the need to bridge biological knowledge and human values in the service of long-term planetary survival (Lecaros, 2025). Health, on this view, is not an exclusively human or biomedical outcome but an emergent property of ecological relationships sustained over time.

Aldo Leopold’s Land Ethic (1949) provides a foundational orientation for this approach by extending moral consideration to the biotic community as a whole. Importantly, subsequent reinterpretations by Callicott (2014) have clarified that Leopold’s ethic should not be read as a call to preserve ‘static ecological equilibrium. Instead, it is compatible with contemporary ecological science, which understands ecosystems as dynamic, adaptive, and historically contingent. Ethical responsibility, from this perspective, lies in sustaining the functional integrity and resilience of socio-ecological systems rather than imposing rigid control.

This interpretation naturally aligns with Leopold’s emphasis on long-term stewardship, in which humans are understood as responsible members rather than managers of the land community. Stewardship, in this sense, requires decisions that account for ecological processes unfolding across generations, not merely short-term efficiency or yield. Land Ethics thus provides a moral framework for sustaining the conditions under which human and non-human life can co-evolve over the long term (Lecaros, 2025).

Applied to One Health governance, global ecological bioethics challenges reactive strategies that prioritize short-term containment without attending to long-term ecological feedbacks. In the context of H5N1, mass culling and crisis-driven interventions may reduce immediate viral load while reinforcing the production systems and land-use practices that generate panzootic vulnerability. Potter’s vision of bioethics as anticipatory wisdom underscores the ethical inadequacy of such approaches, insisting on foresight, precaution and intergenerational responsibility (Ten Have and Gordijn, 2014; Ten Have, 2022).

Despite its relevance, ecological bioethics remains underrepresented within institutional One Health governance and public policy agendas in some regions. International expert bodies and policy platforms continue to be dominated by biomedical and technical expertise, with limited engagement from environmental humanities, political ecology, Indigenous philosophy or social sciences (Rodriguez, 2025). This imbalance constrains the ethical scope of One Health and limits its capacity to address zoonotic risk in ecologically sensitive and culturally diverse contexts.

Integrating global ecological bioethics into One Health governance supports a reflexive model of decision-making, in which ethical deliberation is embedded within surveillance, risk assessment, and intervention design rather than treated as an external constraint. Reflexive governance acknowledges uncertainty, remains open to revision, and responds to ecological feedback and sociocultural learning. As Boudreau LeBlanc *et al.* (2022, 2025) argue, such an approach is essential for addressing complex and evolving challenges such as H5N1 within multispecies socio-ecological systems.

Taken together, zoonoethics and global ecological bioethics provide a structured normative framework—from a micro to a macro-ethical level—that combines a short-term and long-term vision to rethinking One Health governance. Zoonoethics offers ethical sensitivity at sites of multispecies interaction and vulnerability, while ecological bioethics situates these concerns within a planetary, intergenerational context. Their integration enables a shift from reactive biosecurity toward anticipatory, inclusive and ecologically grounded public health ethics—one capable of confronting the structural drivers of panzootic risk and of guiding One Health governance in an era of planetary instability.

## Beyond Culling and Reactive Biosecurity Responses

Across national and regional contexts, the dominant response to H5N1 has relied on large-scale animal culling and emergency biosecurity measures. These interventions are typically justified as necessary to protect public health, secure trade flows and prevent viral amplification within production systems. Yet when examined through a multispecies and socio-ecological lens, mass culling reveals profound ethical, epistemic, and governance limitations. This section advances the central claim of the article: that culling-centered biosecurity constitutes an ethically fragile and structurally insufficient response to panzootic risk, and that H5N1 exposes the need for a deeper normative reorientation of One Health governance grounded in zoonoethics and global ecological bioethics.

### Ethical Limits of Mass Culling and the ‘Poultry Losses’ Narrative

Mass culling has become the default instrument in H5N1 control strategies worldwide, often involving the rapid killing of millions of poultry animals following outbreak detection. While such measures may reduce viral load locally in the short term, they do little to address the structural drivers of viral persistence and spread, particularly in a panzootic context characterized by wildlife reservoirs, migratory flyways and globalized agri-food systems. More importantly, culling raises serious ethical concerns regarding proportionality, necessity and moral justification.

As Degeling *et al.* (2016), Lederman *et al.* (2021), van Herten and Bovenkerk (2021) and van Herten *et al.* (2019) have shown, culling decisions are frequently made under conditions of uncertainty, political pressure and economic urgency, with limited deliberation about alternatives such as vaccination, habitat protection or systemic prevention. Animals are routinely framed as ‘poultry losses,’ ‘stocks,’ or ‘units,’ reflecting a utilitarian calculus that prioritizes economic stability and biosecurity efficiency over animal vulnerability and welfare. This framing aligns with international reporting practices that aggregate deaths from disease and deaths caused by preventive killing into a single category, thereby obscuring the moral distinction between unavoidable harm and deliberate destruction.

Recent ethical scholarship has identified mass culling as a paradigmatic site of unresolved normative conflict within One Health governance. Baard (2025) argues that culling exposes tensions between animal welfare, conservation goals and public health protection that are often treated as technical trade-offs rather than as moral dilemmas requiring explicit justification. While holistic approaches may appeal to collective goods such as ecosystem health or human safety, they frequently do so by normalizing the killing of individual animals without transparent ethical scrutiny. Treating such decisions as matters of coordination rather than ethical deliberation risks entrenching what Baard describes as ‘moral shortcutting,’ thereby undermining the legitimacy of One Health itself (Baard, 2025).

From a zoonoethical perspective, the ethical problem is not merely the scale of killing but the assumptions that render it ethically invisible. The recurrent normalization of culling reflects a deeper unwillingness to confront the food systems, production models and land-use practices that structurally generate zoonotic risk. In this sense, culling functions as a compensatory mechanism: it absorbs the ethical and ecological costs of industrial intensification while leaving its underlying drivers intact. From a global ecological bioethics perspective, the ethical problem lies in rendering invisible the consideration of long-term timelines when making discrete decisions about the risks of zoonotic disease emergence, as well as the lessons learned from the accelerated and changing dynamics and interconnections within socio-ecological systems.

### Selective Stringency and Uneven Distribution of Harm

Ethical concerns are further amplified by the uneven application of biosecurity measures across species and production systems. In poultry governance, ‘stamping-out’ policies remain mandatory in many jurisdictions following detection of H5N1, with strict movement bans and compulsory depopulation. By contrast, regulatory responses to infected dairy cattle in the United States have been markedly less stringent. As Capua and Fanelli (2024) note, cattle farms are subject to temporary movement restrictions and retesting protocols, while guidelines for the disposal of milk and milk products remain largely recommendatory rather than compulsory.

This asymmetry raises important questions of proportionality and moral consistency. Poultry are subjected to immediate and large-scale killing, while cattle—despite evidence of cross-species transmission—are managed through continuity-oriented regimes designed to preserve production. From a zoonoethical standpoint, such selective stringency suggests that biosecurity policies distribute harm not according to ethical principles of vulnerability or risk, but according to economic value, trade sensitivity and political feasibility. The result is a form of multispecies injustice in which certain animal populations are rendered expendable in the service of maintaining industrial systems.

Vaccination policy offers a further illustration of this inconsistency. In several contexts, vaccination against HPAI in poultry remains restricted due to trade and surveillance concerns, reinforcing reliance on culling as the primary control strategy. At the same time, emergency vaccination has been increasingly adopted in parts of Europe and South America, reflecting growing recognition of the limits of depopulation in recurrent outbreak scenarios. These divergences underscore the absence of a coherent ethical framework capable of guiding proportional, species-sensitive responses to multispecies risk.

### Brazil’s H5N1 Outbreak as a Sentinel Event

The ethical fragility of reactive biosecurity became particularly evident with Brazil’s first confirmed outbreak of H5N1 in commercial poultry in May 2025. Occurring within a broader South American panzootic affecting wild birds, poultry and mammals, the outbreak marked a critical shift in the regional epidemiology of the virus. Described as ‘a sentinel event for cross-border preparedness,’ it exposed not only epidemiological vulnerabilities but also the limited capacity of existing governance frameworks to anticipate and manage multispecies risk across interconnected socio-ecological systems (Martins-Filho and Quintans-Júnior, 2025).

While emergency measures succeeded in containing the immediate outbreak, they did so by reproducing the familiar logic of rapid depopulation and trade-oriented reassurance. From a zoonoethical perspective, this response illustrates how emergency biosecurity protocols prioritize speed and containment at the expense of ethical deliberation, transparency and long-term prevention. Decisions involving large-scale animal killing were implemented without robust procedures for ethical oversight or public justification, reinforcing the perception that such harms are inevitable rather than contestable.

Moreover, Brazil’s response highlights how national outbreak management can externalize harms across ecological and political boundaries. Migratory species, wildlife populations and rural communities bear cumulative costs that are rarely captured within national accounting frameworks. This externalization reflects a broader pattern in which reactive biosecurity treats outbreaks as isolated emergencies rather than as indicators of systemic imbalance requiring structural intervention.

### Emergency Ethics, Short-Termism and the Governance of Crisis

The dominance of emergency ethics in H5N1 governance reflects a deeper failure to adopt long term, adaptive modes of moral reasoning. Drawing on Aldo Leopold’s Land Ethic (1949), ethical action should be evaluated in terms of whether it preserves the ‘integrity, stability, and beauty of the biotic community.’ As has been shown (Van Herten and Bovenkerk, 2021), reactive biosecurity strategies grounded in rapid elimination and short-term risk suppression sit uneasily with an ethical criterion that demands the long-term evaluation of risk and containment practices. Large-scale culling not only disrupts complex ecological relations, but also reproduces the very high-density production regimes that generate pathogenic vulnerability, while simultaneously diverting ethical and political attention away from preventive approaches such as habitat protection, landscape-level surveillance, and differentiated, context-sensitive interventions.

From the perspective of adaptive governance, repeated reliance on culling forecloses opportunities for learning and revision. Treating outbreaks as discrete emergencies encourages a cycle of response that privileges immediate control over ecological understanding, thereby increasing long-term vulnerability to H5N1 and similar zoonotic threats. Zoonoethics challenges this logic by calling for a shift from an ethics of domination to an ethics of reflexive participation and constructive dialogue—one that recognizes humans as members of a biotic community rather than as unilateral managers of life and death.

In this view, ethical governance requires moving beyond the assumption that large-scale animal killing is a morally neutral instrument of crisis management. Instead, it demands sustained ethical scrutiny of how interventions shape multispecies relations over time and whether they align with principles of interspecies justice, ecological stewardship and anticipatory care (Raymond *et al.*, 2025). Only by embedding such reflection within One Health governance can responses to H5N1 move beyond episodic containment toward strategies capable of reducing vulnerability at the interfaces of human, animal and environmental health.

## Ethical Guidelines for Inclusive and Context-sensitive One Health Governance

**Figure 1.**
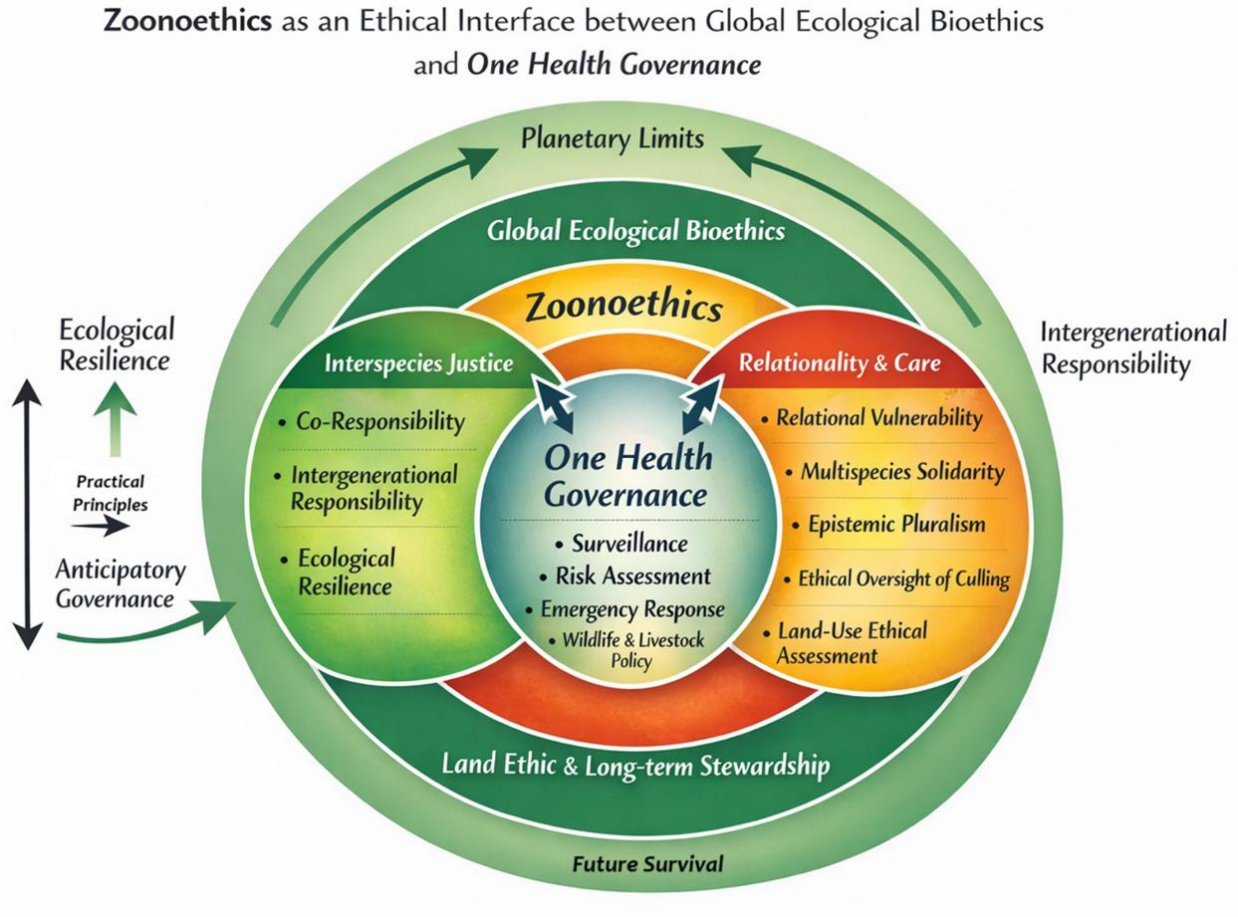
Zoonoethics as an ethical interface between global ecological bioethics and One Health governance.

In light of the ethical shortcomings identified in prevailing biosecurity-centered responses to H5N1, there is a need for a coherent normative framework capable of guiding One Health governance beyond reactive containment. The principles outlined below do not function as operational rules or policy prescriptions, but as ethical orientations intended to structure deliberation, accountability and institutional design in contexts of multispecies risk. Grounded in zoonoethics and global ecological bioethics, they articulate the moral commitments required to govern zoonotic threats in ways that acknowledge relational vulnerability, multispecies justice, and shared responsibility across ecological and social scales (Figure 1).

This diagram does not represent a Venn model of overlapping domains, but a layered structure of ethical complexity. Global ecological bioethics provides the planetary normative horizon, grounding governance in long-term ecological limits and intergenerational responsibility. Zoonoethics operates as an interfacial and action-guiding ethical framework, translating these commitments into practical principles for decision-making at human–animal–ecosystem interfaces. One Health governance emerges from the interaction between these layers as an ethical–political project of multispecies care, rather than as a purely technical or biomedical tool. Within this layered structure, the concept of planetary boundaries frames the biophysical thresholds that condition ethical action, making long-term survival a normative constraint rather than a secondary objective (Rockström *et al.*, 2024; Gidden *et al.*, 2025). Respecting these limits situates ethical deliberation within a finite Earth system, where exceeding ecological thresholds undermines multispecies flourishing. Long-term survival thus becomes an ethical criterion that links planetary governance, interspecies responsibility and the durability of socio-ecological systems over time.

(i) *Interspecies Justice*: Zoonoethics begins from the recognition that farmed, wild and companion animals possess intrinsic value and ethical significance that cannot be reduced to their instrumental roles in human health, economic productivity or epidemiological control (Batavia and Nelson, 2017; Owe *et al.*, 2022). Interspecies justice challenges weak anthropocentric frameworks that treat animal suffering as ethically relevant only insofar as it affects human well-being. Within One Health governance, this principle requires that interventions such as mass culling be subjected to explicit ethical justification, rather than assumed as morally neutral necessities. The large-scale killing of poultry and wildlife in response to H5N1 illustrates how animals are routinely positioned as expendable units within biosecurity systems, raising fundamental questions about multispecies justices, proportionality and moral exclusion (Celermajer and McKibbin, 2023; Celermajer *et al.*, 2023).

(ii) *Relational Vulnerability:* Rather than conceiving vulnerability as an individual deficit, zoonoethics understands vulnerability as a relational condition emerging from ecological and social interdependence. As Rodriguez argues, multispecies life is characterized by ‘embodiment and relational vulnerability’ (Rodriguez, 2025, p. 331). In the context of H5N1, this perspective reframes outbreaks as shared crises affecting animals, humans, ecosystems, and livelihoods simultaneously. Ethical governance must therefore assess risk and harm systemically, moving beyond anthropocentric metrics that prioritize human exposure while rendering nonhuman suffering ethically peripheral.

(iii) *Co-Responsibility and Shared Ethical Accountability:* Zoonoethics assigns heightened ethical responsibility to humans and institutions that disproportionately shape ecological conditions through deforestation, industrial farming, extractive economies and climate change. Co-responsibility extends beyond individual actors to encompass states, corporations, international organizations and digital governance regimes, whose decisions actively structure conditions of zoonotic vulnerability (Hendl and Shukla, 2024; Verhenneman, 2025; Gao *et al.*, 2026).

In the case of H5N1, this principle underscores that effective response cannot be limited to emergency containment but must include accountability for the socio-ecological disruptions that make panzootics increasingly likely. Co-responsibility thus reframes prevention as a collective ethical obligation rather than a matter of technical risk management.

(iv) *Stewardship and Multispecies Solidarity:* Drawing on global ecological bioethics and Indigenous philosophies, zoonoethics conceptualizes stewardship as a practice of care, humility and guardianship toward the more-than-human world. This orientation resonates with Leopold’s Land Ethic and with biocultural approaches that emphasize the biocultural conservation of interconnected habitats, practices and communities (Rozzi, 2015). Multispecies solidarity further extends this commitment by recognizing animals not as adversaries in a ‘war against disease,’ but as co-inhabitants of shared socio-ecological systems (Gruen, 2024). In the H5N1 context, this principle invites critical reflection on both reactive culling policies and the routine violence of industrial animal agriculture, which together normalize animal disposability and undermine long-term ecological resilience (Rodriguez, 2024, 2025).

(v) *Epistemic Pluralism and Intercultural Knowledge:* Both zoonoethics and global ecological bioethics insists that scientific and technical expertise, while indispensable, is insufficient on its own to govern complex zoonotic risks. Indigenous knowledge systems, smallholder farmers’ experiential expertise, and local ecological understandings provide critical insights into environmental change, wildlife behavior, and early warning signals (Redvers *et al.*, 2022 ; Pollowitz *et al.*, 2024). Epistemic pluralism challenges governance structures that marginalize non-Western and non-scientific knowledge, often reproducing patterns of epistemic injustice (Bingham *et al.*, 2025; Redvers *et al.*, 2025). In One Health governance, this principle demands institutional mechanisms that integrate diverse forms of knowledge as co-constitutive of decision-making, rather than as consultative add-ons.

(vi) *Structural–Ecological One Health Governance:* Zoonoses such as H5N1 are not discrete biological events but systemic phenomena shaped by interrelated environmental, social and economic forces (Rodriguez, 2024). This principle shifts the focus of governance from pathogens to the structural conditions that generate vulnerability, including land-use change, biodiversity loss, agro-industrial intensification, and climate instability. Structural–ecological governance requires that One Health responses address upstream drivers of zoonotic risk, rather than relying exclusively on downstream containment measures. From an ethical standpoint, this approach aligns with global ecological bioethics by situating health within planetary limits and socio-ecological feedback loops (Lecaros, 2016a; Valdés and Lecaros, 2023).

(vii) *Ethical Oversight of Culling and Emergency Measures:* The normalization of mass culling as an emergency response raises urgent ethical concerns regarding proportionality, precaution and long-term harm. Zoonoethics calls for formal ethical oversight mechanisms to evaluate interventions such as culling, carcass disposal and movement restrictions before crises occur. Empirical studies documenting risks associated with burial practices—such as groundwater contamination and wildlife exposure—highlight how reactive decision-making can generate new ecological vulnerabilities (Figueroa *et al.*, 2021; Souillard *et al.*, 2025). Ethical oversight must therefore extend beyond immediate epidemiological goals to assess cumulative and multispecies impacts over time.

(viii) *Intergenerational Responsibility*: Zoonotic risk is cumulative and intergenerational: ecological harm inflicted today shapes the vulnerability of future human and non-human communities. Global ecological bioethics incorporates obligations to future generations by emphasizing long-term ecological viability and the preservation of multispecies relationships. In the context of H5N1, intergenerational responsibility calls into question short-term crisis management strategies that externalize ecological, social and health costs onto future generations. It reinforces the need for anticipatory governance grounded in global ecological bioethics, which provides a normative and legal horizon oriented toward precaution, sustainability and the systematic anticipation of long-term risks (Lecaros, 2013; 2016a).

(ix) *Reforming Intensive Livestock Systems:* A growing body of evidence identifies intensive livestock production as a major amplifying environment for zoonotic spillover (Hage *et al.*, 2024; Ahmed *et al.*, 2025). Zoonoethics therefore supports a normative shift away from high-density, industrial animal farming toward agroecological and low-density systems that reduce multispecies vulnerability. This principle does not frame dietary or production transitions as moral imperatives imposed from above, but as ethically justified responses to structural risk, grounded in interspecies justice, ecological responsibility, and public health protection.

(x) *Integrating Ecological and Socio-Sanitary Land-Use Assessment:* Finally, zoonoethics emphasizes the integration of ecological and socio-sanitary land-use assessment into One Health governance. Land transformation, habitat fragmentation and expanding agricultural frontiers restructure human–animal–environment relations, creating predictable conditions for zoonotic emergence (Sell and Williams, 2020; Keesing and Ostfeld, 2021). Ethical governance must therefore evaluate how land-use decisions distribute risk across species and communities, embedding health considerations within environmental planning and territorial governance. This principle operationalizes a global ecological bioethics by linking planetary processes to site-specific decision-making.

Taken together, these 10 principles articulate a zoonoethical framework for One Health governance that moves beyond reactive biosecurity toward anticipatory, inclusive and ecologically grounded public health ethics. By integrating multispecies justice, relational vulnerability and shared responsibility, zoonoethics complements global ecological bioethics in reimagining One Health as a normative project of multispecies cohabitation. In the face of recurrent panzootics such as H5N1, such a reorientation is not only ethically desirable but increasingly necessary for legitimate and effective public health governance.

## Discussion: A Perfect Panzootic Storm for One Health Governance

The H5N1 panzootic exposes what may be described as a ‘perfect storm’ for One Health governance: the convergence of ecological disruption, industrial animal production, fragmented surveillance systems and ethical under-theorization. As the preceding analysis has shown, dominant responses—particularly mass culling and reactive biosecurity—have achieved limited success in producing short-term containment at specific sites, yet they systematically fail to address the structural conditions that generate recurrent multispecies risk. These failures are not merely operational or technical; they reflect deeper normative blind spots within One Health, where ethical reasoning remains largely subordinated to emergency logics, trade imperatives and technocratic expertise (Lederman and Capps, 2020; Boudreau LeBlanc *et al.*, 2022, 2025; Capps, 2022, 2024, 2025).

At the governance level, the H5N1 panzootic reveals a persistent gap between the multispecies aspirations of One Health and its institutional practices. While the framework formally acknowledges interdependence among human, animal and environmental health, its implementation continues to externalize ecological harm, normalize large-scale animal killing and marginalize non-Western epistemologies. Structural drivers of zoonotic emergence—such as deforestation, agro-industrial expansion, habitat fragmentation and the displacement of Indigenous and local communities—remain largely invisible within formal risk assessments, despite their well-documented role in amplifying spillover events. This omission points to a critical limitation of prevailing governance models: the absence of a robust ethical account of responsibility for upstream, politically and economically mediated causes of disease.

From the perspective of public health ethics, H5N1 also brings into sharp relief unresolved tensions within One Health itself. Principles such as stewardship, biodiversity protection, and animal welfare are frequently invoked in policy discourse, yet they are routinely overridden in practice by short-term emergency ethics and short-term calculations. Conflicts between conservation goals, food security, economic interests and multispecies justice are treated as technical trade-offs rather than as moral dilemmas requiring transparent justification and public accountability. As a result, decisions involving life and death—particularly those affecting farmed and wild animals—are made with limited ethical scrutiny, reinforcing an anthropocentric posture in which humans act as privileged arbiters rather than as participants in a shared biotic community.

Zoonoethics clarifies why these failures persist and what remains unresolved in current approaches to One Health governance. By foregrounding interspecies justice, relational vulnerability, co-responsibility and epistemic pluralism, zoonoethics exposes the limits of governance models that prioritize control over care and efficiency over legitimacy. The Brazilian case illustrates how reactive biosecurity externalizes harm across species and generations, while obscuring the normative choices embedded in outbreak response. More broadly, the H5N1 panzootic raises pressing ethical questions that extend beyond this specific case: How should responsibility be allocated when zoonotic risks are produced by global systems rather than localized failures? How can Indigenous and local knowledge systems be integrated as co-governing forces rather than consultative supplements? And how can One Health shift from crisis-driven intervention toward anticipatory stewardship of multispecies health?

Addressing these questions requires reconceptualizing One Health not as a technical toolkit but as an ethical and political project situated within planetary constraints. Global ecological bioethics contributes to this reconceptualization by emphasizing reflexivity, precaution and intergenerational responsibility, while zoonoethics operationalizes these commitments at sites of multispecies interaction and vulnerability. Together, they offer a normative framework capable of guiding governance in contexts characterized by uncertainty, complexity and structural injustice. Without such a reorientation, One Health risks reproducing the very conditions it seeks to mitigate, responding to panzootics through cycles of emergency management that leave underlying drivers intact.

## Conclusion

The H5N1 panzootic cannot be understood solely as an epidemiological crisis; it constitutes an ethical, ecological and political threshold that reveals the depth of interdependence among humans, animals and ecosystems. This article has argued that prevailing responses, centered on mass culling and reactive biosecurity, are ethically insufficient and structurally misaligned with the multispecies complexity of contemporary panzootics. By treating animal death as a technical instrument of disease control and by privileging short-term containment over structural prevention, current governance models fail to address the conditions that make zoonotic crises increasingly frequent and severe.

Drawing on zoonoethics and global ecological bioethics, the article has proposed a reconfiguration of One Health governance grounded in interspecies justice, relational vulnerability, shared responsibility and epistemic pluralism. Through the case of Brazil’s response to H5N1, it has shown how reactive strategies obscure ethical accountability and marginalize both non-human life and the human communities most affected by ecological disruption. In contrast, a zoonoethical approach reframes One Health as a project of multispecies cohabitation, requiring anticipatory governance, ethical oversight of emergency measures, and sustained attention to structural drivers such as land-use change, industrial livestock production, and environmental injustice.

For public health ethics, the implications of this analysis are significant. Legitimate and effective governance of panzootic risk demands more than improved surveillance or faster response; it requires ethical frameworks capable of guiding decisions about whose lives are protected, whose harms are normalized, and how responsibility is distributed across species, societies and generations. In an era of planetary instability and recurrent zoonotic threats, reimagining One Health as an ethical and political undertaking is not an aspirational add-on but a practical necessity. Only by embedding multispecies ethics within public health governance can responses to crises such as H5N1 move beyond reactive control toward more just, resilient and ecologically coherent forms of collective care.
