# Correction to: Zoonoethics and Inclusive One Health Governance for H5N1 Panzootic: From Animal Culling to Co-responsibility

**DOI:** 10.1093/phe/phag007

**Published:** 2026-05-06

This is a correction to: Juan Alberto Lecaros, Jeyver Rodriguez, Zoonoethics and Inclusive One Health Governance for H5N1 Panzootic: From Animal Culling to Co-responsibility, *Public Health Ethics*, Volume 19, Issue 1, April 2026, phag002, https://doi.org/10.1093/phe/phag002

In the originally published version of this manuscript, one of author, Jeyver Rodriguez’s, affiliations was noted as, Center for Socio-Legal, Criminological, and Ethical Research (CISCE), Universidad Central, Santiago de Chile, Chile

This should be Center for Socio-Legal, Criminological, and Ethical Research (CISCE), Universidad Central de Chile, Santiago de Chile, Chile

This error has been corrected.

